# Brief treatments for panic disorder: a pilot study of the bergen repeated one-session treatment format

**DOI:** 10.1186/s12888-025-07499-4

**Published:** 2026-01-07

**Authors:** Bjarne Hansen, Kay Morten Hjelle, Thröstur Björgvinsson, Olav Erland, Kristen Hagen

**Affiliations:** 1 Bergen Center for Brain Plasticity, Haukeland University Hospital, Bergen, Norway; 2 Center for Crisis Psychology, Faculty of Psychology, University of Bergen, Bergen, Norway; 3 McLean Hospital, Belmont, USA; 4 Harvard Medical School, Boston, USA; 5 Department of Psychiatry, Møre and Romsdal Hospital Trust, Molde, Norway; 6 Department of Mental Health, Norwegian University of Science and Technology, Trondheim, Norway

**Keywords:** Panic disorder, CBT, Brief, Exposure, BrOST

## Abstract

**Background:**

Panic disorder (PD) significantly impacts individuals’ daily lives and overall well-being. While cognitive behavioral therapy (CBT) is the gold standard for PD treatment, there is a growing interest in brief interventions to improve accessibility and reduce treatment burden. This study explored the feasibility and preliminary outcomes of a brief, four-session CBT intervention for PD, grounded in an intention-focused approach. The treatment emphasized modifying patients’ intentions underlying avoidance and safety behaviors during exposure tasks, aiming to enhance engagement and therapeutic effect.

**Methods:**

This naturalistic effectiveness study involved 27 patients from the Norwegian Specialist Health Care clinic who were referred for PD treatment. Participants received the Bergen Repeated One-Session Treatment (BrOST), a brief, intention-focused cognitive behavioral intervention delivered in four individual sessions over a period of two to three weeks. Standardized self-report measures were administered to assess the severity of panic symptoms, general anxiety, depressive symptoms, and treatment satisfaction.

**Results:**

The treatment resulted in significant reductions in PD severity from pretreatment to one week posttreatment, with sustained improvements at three months. There were also notable reductions in symptoms of anxiety and depression. High levels of treatment satisfaction were reported by the patients. These findings suggest that the BrOST could be a promising alternative to traditional CBT protocols.

**Conclusion:**

This study indicated that the BrOST may be a promising brief CBT intervention for individuals with PD. Larger and more controlled studies are warranted.

**Trial registration:**

The study was reviewed by the Regional Committee for Medical Research Ethics Northern Norway, REK North (REK Midt 2023/ 701114).

## Introduction

Panic disorder (PD) is a debilitating condition marked by unexpected and severe panic attacks that significantly impacts individuals’ daily lives and overall well-being. It is estimated that PD affects approximately 1 to 4% of the global population, highlighting it as a significant public health issue [1]. Cognitive behavioral therapy (CBT) is recognized as the gold standard for PD treatment, as endorsed by clinical guidelines and supported by extensive research, notably by the National Institute for Health and Care Excellence [2].

Traditionally CBT protocols for PD patients involve 11 to 18 sessions delivered over several months [3]. However, there is an increasing trend toward condensed treatment formats aimed at improving accessibility, reducing treatment burden and enhancing cost-effectiveness [3]. Although the definition of ‘brief treatment’ lacks a consistent definition, Öst and Ollendick have suggested that it refers to interventions delivered in markedly fewer sessions than conventional treatment formats [3]. A growing body of research supports the potential of such approaches. For example, Varma [4] reported that 35% of participants had a panic-free status after just three sessions focusing on psychoeducation and hyperventilation control. Similarly, Westling and Öst [5] reported a 90% success rate in eradicating panic attacks with a four-session protocol. Craske and colleagues found that 38% of patients who underwent four sessions of CBT showed sufficient improvement to withdraw from a pharmacological trial and/or no longer met the criteria for PD by the end of the treatment. This outcome was in contrast to non directive supportive therapy, which did not yield significant effects [6]. Furthermore, comparative studies have shown minimal differences in effectiveness between five-session treatments and traditional longer CBT protocols, supporting the potential benefits of these brief interventions [7]. Additionally, the incorporation of pharmacological agents such as d-cycloserine with a five-session CBT regimen was reported to significantly improve PD symptoms [8, 9].

Despite these promising developments, few studies on brief treatment formats for PD have applied standardized diagnostic procedures, used validated measures such as the PDSS, or included systematic follow-up. As a result, the evidence base remains limited, and further evaluation of these approaches is warranted.

The Bergen repeated one-session treatment (BrOST) is a development from the Bergen 4-day treatment (B4DT) which is a group based treatment delivered in 4-consecutive days. The B4DT have showed promising results for OCD [10–20], PD [21–27] and SAD [28] BrOST shares the same theoretical foundation as B4DT, emphasizing intention-focused CBT as operationalized in the “Leaning into Anxiety” (LET) protocol. This approach centers on the deliberate examination and modification of the intentions that underlie both exposure behavior and safety strategies. While B4DT is delivered over four consecutive days in a group setting and typically involves approximately 21 h of therapist contact per patient, BrOST restructures the intervention into four individual sessions delivered across two to three weeks, along with two optional follow-up sessions. The total therapist contact time in BrOST is approximately six hours.

BrOST was developed to preserve the core therapeutic principles of B4DT while offering a format that is more flexible and resource-efficient. Delivered individually over a brief period, the intervention allows for greater adaptability within standard outpatient services. In contrast to standard CBT protocols for PD, which typically emphasize symptom-focused exposure and cognitive restructuring to correct catastrophic misinterpretations [29], BrOST focuses on shifting the patient’s intentional stance toward anxiety by encouraging active and willing engagement with feared experiences. This process-oriented approach facilitates exposure work that can be individualized to match each patient’s specific patterns of avoidance and engagement.

The aim of the study was to evaluate the feasibility and preliminary effects of the BrOST intervention for individuals with PD. We hypothesized that the intervention would lead to significant reductions in panic symptoms, as measured by the PDSS, from pre- to post-treatment and at follow-up. We further hypothesized that the intervention would reduce comorbid symptoms of anxiety and depression and result in high levels of treatment satisfaction.

## Methods

### Participants and procedures

The present study was a naturalistic investigation of 27 patients receiving outpatient treatment at the publicly funded Norwegian specialist mental healthcare clinic. The intervention was delivered as part of ordinary care. Patients could continue pre-existing psychological or medical care, but no new psychological interventions were initiated during the BrOST treatment period. Medication use remained stable throughout. After the treatment period, patients were free to receive follow-up care or support for comorbid conditions as needed.

To increase treatment availability, patients referred to the clinic for the 4-Day Treatment program (Norway) between February 2022 and November 2023 were offered brief CBT (4 sessions) for PD. We used the Mini International Neuropsychiatric Interview (MINI) to diagnose PD and to screen for comorbid disorders [30], as the Norwegian version of the interview has reasonable psychometric properties [31]. According to the MINI, 16 patients had PD with agoraphobia (59.3%), and 11 patients had PD without agoraphobia (40.7%). Patients were not included if they were in active psychosis or mania, had a severe eating disorder, or were at high risk of self-injury or suicide.

An overview of the demographic variables is provided in Table 1. Twenty-one (77.8%) of the patients were female. The mean age of the participants was 30.48 years (range 19–64 years, SD = 11.7), and the average duration of PD was 70.37 months (range 7–334 months, SD = 74.2). Comorbidities included current depressive episodes (*n* = 7, 25.9%), social anxiety disorder (*n* = 3, 11.1%), generalized anxiety disorder (*n* = 5, 18.5%) and recent depressive episodes (*n* = 2, 7.4%). Ten patients (37.0%) were currently on selective serotonin reuptake inhibitors (SSRIs), and only one patient (3.7%) reported sporadic use of benzodiazepines. Patients were advised to abstain from benzodiazepine use during the treatment period, while no changes were made to the use of SSRIs.

**Table 1:** Demographic variables and clinical characteristics

**Variable**	M (SD)
Age (years)	30.48 (11.76)
Duration of PD (months)	70.37 (74.21)
	N (%)
Sex: female	21 (77.8)
Have received previous treatment	17 (63.0)
Marital status	
Single	12 (44.4)
Married or cohabiting	13 (48.4)
Partnered (not cohabiting)	2 (7.4)
Caring for children	6 (22.2)
Educational status	
Junior high school	2 (7.4)
High school	10 (37.0)
College	15 (55.5)
Work status	
Working/studying full time	18 (66.7)
Working part time/unemployed	2 (7.4)
Sick leave/welfare	7 (25.9)
PD with agoraphobia	16 (59.3)
Other comorbidity	14 (51.9)
Comorbid anxiety	8 (29.6)
Comorbid depression	7 (25.9)
Psychotropic medication	11 (40.7)
SSRIs/SNRIs	10 (37.0)
Benzodiazepines	1 (3.7)

### Assessment and measures

Data on the primary outcome measure (the Panic Disorder Severity Scale; PDSS [32] were collected through a clinical interview before treatment and one week and three months after treatment. Additional questionnaires covering symptoms of depression, generalized anxiety were also administered at these three time points. Patients who did not complete the self-reports were reminded by telephone and during the follow-up sessions.

The PDSS [32] were administered as an interview by clinicians. The PDSS includes seven questions targeting different aspects of PD and is found to have adequate psychometric properties for measuring the severity of PD symptoms [33]. To define response and remission, we applied the criteria defined by Furukawa et al. [34]; response was defined as a reduction in the PDSS score of 40%, and remission was defined as a PDSS score of seven points or less in patients with agoraphobia and five points or less in patients without agoraphobia [34]. According to Furukawa [34], we categorized patients as follows based on the change in their PDSS score after treatment: “very much improved” (75–100% reduction), “much improved” (40–74% reduction), “minimally improved” (10–39% reduction) or “no improvement” (0–10% reduction).

Symptoms of depression were measured with the Patient Health Questionnaire (PHQ-9) [35], a nine-item questionnaire (scores range from 0 to 27 points). Symptoms of generalized anxiety were measured with the Generalized Anxiety Scale (GAD-7) [36], which is a seven-item questionnaire (scores range from 0 to 21 points). To investigate treatment satisfaction, we applied the Client Satisfaction Questionnaire (CSQ-8) [37]. The CSQ-8 is an eight-item questionnaire (scores range from 8 to 32 points) and was administered one week after treatment.

### Treatment

Patients received a brief, intention-focused cognitive behavioral intervention for panic disorder, based on a structured manual developed by the first author. The format consisted of four individual sessions delivered over a period of two to three weeks, typically with two sessions per week. The first two sessions lasted between 90 and 150 min, and the final two sessions between 45 and 60 min, resulting in approximately six hours of therapist contact time in total.

The full treatment rationale was introduced in the first session and revisited in each of the subsequent sessions to consolidate understanding. Each session included psychoeducation, in-session interoceptive exposure (e.g., hyperventilation, stair running), and in vivo exposure exercises when feasible. Patients were also guided in planning and reflecting on between-session tasks.

In vivo exposure was conducted both during sessions and assigned as structured homework between sessions. These tasks were individually tailored to each patient’s avoidance patterns and often involved agoraphobic situations such as using public transport, waiting in line, or entering crowded environments. The aim was to help patients apply the treatment principles in real-world settings, with experiences reviewed and discussed in subsequent sessions.

A central focus of the intervention was to shift the patient’s intentional stance toward anxiety symptoms. Based on the LET protocol, the treatment emphasized active engagement over avoidance, with the goal of helping patients relate differently to their bodily sensations. Rather than focusing on reducing symptoms or achieving habituation, patients were guided to approach anxiety symptoms as harmless signals that had previously misled them into avoidance. Exposure tasks were often repeated within and across sessions until the patient demonstrated a clear understanding of this principle and an intentional shift in behavior. A central tenet was that it is more meaningful to complete a small task with the right focus than a more difficult task driven by avoidance or safety behaviors.

Patients were encouraged to take an active role in selecting and shaping exposure tasks, fostering a sense of autonomy and personal relevance. The overarching goal of treatment was not symptom elimination, but rather to reduce the degree to which panic disorder constrained everyday functioning. This emphasis on intentional engagement and behavioral flexibility distinguishes BrOST from standard CBT protocols for panic disorder, which more often focuses on reducing catastrophic misinterpretations of bodily sensations through cognitive restructuring and symptom-driven exposure [29].

All patients were offered two follow-up sessions: one approximately one week after treatment and one three months later. These sessions were used to reinforce the rationale, address challenges, and discuss relapse prevention strategies. As the study was conducted in a routine care setting, some patients also received additional follow-up. In total, five patients (18.5%) received one or more extra sessions (mean = 1.2, range = 1–4; 45 min each).

The overall treatment structure was consistent across patients, but the focus of psychoeducation and choice of exposure were individualized. Patients varied in the somatic symptoms they feared (e.g., dizziness, heart palpitations), the catastrophic interpretations they held (e.g., fear of going insane or fainting), and the situations they avoided. For example, a patient who feared fainting in supermarkets was guided to enter such environments and remain present without engaging in avoidance or safety behaviors, while adopting a stance of willingness toward the feared sensations. Exposure tasks were chosen to reflect what mattered most to each individual and to directly address the obstacles that interfered with how they wanted to live their lives.

### Therapists

The therapists who participated in the treatment were clinical psychologists at the Clinic for 4-day Treatment at Haukeland University Hospital. A total of ten therapists were involved in the treatment, with an average of 3.41 years of experience in the treatment of anxiety disorders (range 2–6 years). As the treatment was part of ordinary clinical care, no measure of treatment fidelity was applied. The therapists were supervised by the first and the last author of the paper with 15.5 years of experience in the treatment of anxiety disorders (range 14–17 years).

### Statistical analysis

This was an exploratory pilot study, and no formal power analysis was conducted. A target sample size of approximately 30 participants was chosen based on recommendations for pilot studies, where samples of this size are often considered sufficient to assess feasibility and estimate variability [38]. Comparable sample sizes have been used in pilot trials of the B4DT, which conceptually underpins the current intervention. Previous studies of B4DT for panic disorder have demonstrated large effect sizes, supporting the expectation that meaningful pre-post changes could be observed even in a modest sample. There were no dropouts during the treatment period.

IBM SPSS statistics (version 29) was used for all analyses. For the clinical interviews (PDSS), there were no missing data before treatment or at one week or three months after treatment. Regarding self-reports, five patients (18.5%) had missing data before treatment, four patients had missing data at one week (14.8%), and seven patients (25.9%) had missing data at three months. To handle the missing data before applying ANOVA, we applied the SPSS expectation maximization method. The expectation maximization method is considered a suitable method for replacing missing data when less than 25% of the total data points are missing (Little’s MCAR test: *χ²(*101) = 102.81, *p* =.431) [39]. Repeated measures ANOVA was conducted for the PDSS, GAD-7 and PHQ-9 using data from all patients. Paired samples t-tests were used for post hoc comparisons between time points. No correction for multiple comparisons was applied, as the study was exploratory and based on a small sample. This approach was intended to balance the risk of Type I and Type II errors in the context of a pilot trial. Cohen´s d (*M* pretreatment – *M* posttreatment)/*SD* pooled) was used to calculate the effect size for the change over the three time points [40].

## Results

### Clinical outcomes

There was a significant reduction in panic disorder symptoms over time, *F*(2, 52) = 83.96, *p* <.001, partial *η²* = 0.76. PDSS scores decreased from 15.00 (SD = 3.01) at baseline to 5.19 (SD = 4.29) at posttreatment and 4.26 (SD = 5.01) at three-month follow-up. Pairwise comparisons showed significant reductions from pretreatment to posttreatment (*p* <.001, *d* = 2.65) and from pretreatment to follow-up (*p* <.001, *d* = 2.60), with no significant change between posttreatment and follow-up (*p* =.607), indicating that treatment gains were maintained.

Generalized anxiety symptoms, as measured by the GAD-7, also declined significantly over time, *F*(2, 52) = 8.05, *p* <.001, partial *η²* = 0.24. Scores decreased from 9.22 (SD = 5.22) at baseline to 6.26 (SD = 5.73) at posttreatment and 5.47 (SD = 4.28) at follow-up. A significant reduction was observed from pretreatment to follow-up (*p* =.004, *d* = 0.79), whereas the change from pretreatment to posttreatment did not reach statistical significance (*p* =.065, *d* = 0.54). No significant difference was found between posttreatment and follow-up (*p* =.593).

Depressive symptoms, measured by the PHQ-9, also showed a significant overall reduction, *F*(2, 52) = 4.73, *p* =.013, partial *η²* = 0.15. Mean scores declined from 9.44 (SD = 5.38) at baseline to 6.96 (SD = 5.55) posttreatment and 6.28 (SD = 2.88) at follow-up. A significant reduction was found from pretreatment to follow-up (*p* =.025, *d* = 0.73), while the reduction from pretreatment to posttreatment did not reach statistical significance (*p* =.152, *d* = 0.45). No significant change was observed between posttreatment and follow-up (*p* = 1.000).

Treatment satisfaction, as measured by the CSQ-8 at follow-up, was high, with a mean score of 28.9 (maximum = 32), indicating that participants were generally very satisfied with the treatment.

A full summary of means, standard deviations, p-values, and effect sizes is presented in Table 2.

**Table 2:** Results (M and SD) for the primary and secondary outcome measures

Measure	Pre M (SD)	Post M (SD, *p*, d)	Follow-up M (SD, *p*, d)	
PDSS	15.00 (3.01)	5.19 (4.29, < 0.001, d = 2.65)	4.26 (5.01, < 0.001, d = 2.60)	
GAD-7	9.22 (5.22)	6.26 (5.73, 0.065, d = 0.54)	5.47 (4.28, 0.004, d = 0.79)	
PHQ-9	9.44 (5.38)	6.96 (5.55, 0.152, d = 0.45)	6.28 (2.88, 0.025, d = 0.73)	
CSQ-8		28.9		

*PDSS* Panic Disorder Severity Scale, *GAD-7* Generalized Anxiety Disorder-7, *PHQ-9* Patient Health Questionnaire-9, *CSQ-8* Client Satisfaction Questionnaire- 8

*p*-values refer to pairwise comparisons versus baseline (Pre). Effect sizes are Cohen’s d, calculated for Pre–Post and Pre–Follow-up, respectively

### Treatment satisfaction

Treatment satisfaction was assessed using the CSQ-8. As shown in Table 3, the mean CSQ-8 score one week after treatment was 28.9 (SD = 3.87), indicating a high level of reported satisfaction. For comparison, a recent Norwegian validation study of the digital CSQ-8, conducted in routine specialist outpatient mental health services and using the same electronic data collection method, reported a lower mean score of 23.67 (SD = 6.08) [41].

**Table 3:** Patient scores on the client satisfaction Questionnaire-8 (CSQ-8)

Question	Mean score	Frequency of each score			
		1	2	3	4
1. Quality of service	3.52	0	3	4	14
2. Kind of service	3.43	0	2	8	11
3. Met needs	3.43	0	2	8	11
4. Recommend to a friend	3.90	0	0	2	19
5. Amount of help	3.48	1	1	6	13
6. Deal with problems	3.71	0	1	4	16
7. Overall satisfaction	3.67	0	1	5	15
8. Come back	3.71	0	1	4	16

## Discussion

This study explored the feasibility and preliminary outcomes of the BrOST, a brief four-session treatment protocol for individuals with PD. A growing body of research supporting brief interventions, as demonstrated by Varma [4] and Westling & Öst [5], suggests that a reduced duration of traditional CBT can still yield significant therapeutic benefits without compromising efficacy. Our findings are in line with these studies, providing additional evidence that brief, targeted interventions can lead to substantial reductions in PD symptoms.

This study contributes to the literature by applying a brief CBT approach emphasizing the modification of intentions behind exposure and safety-seeking behaviors rather than focusing on the exposure itself. In addition to symptom reduction, our study emphasizes understanding and altering the underlying intentions, providing a potentially effective treatment for PD. This is in line with the outcomes observed in the B4DT, which has shown promising results in a series of studies using similar principles [17, 20, 24, 26–28]. The key difference between the two treatments lies in their delivery, as BrOST was provided individually and requires significantly less therapist time compared to the more comprehensive, group-based B4DT.

Our results showed a significant reduction in PDSS scores from pretreatment to posttreatment, with sustained improvements at the three-month follow-up. This indicates that the BrOST might be a promising and cost-effective approach for PD patients. The results are also comparable to the limited numbers of previous studies on brief treatments for PD. In addition, the reductions in symptoms of anxiety and depression indicate that the treatment may also be effective for secondary symptoms. The high levels of patient satisfaction indicate that treatment may be feasible for patients with PD.

While traditional CBT protocols for PD span 11 to 18 sessions [3], our findings suggest that a brief CBT format can achieve comparable outcomes. By reducing time and resource commitments, brief interventions such as the BrOST have the potential to address barriers to treatment access and adherence, offering a promising approach for the undertreatment of PD [42].

The high level of treatment satisfaction observed in the present sample suggests that the BrOST intervention was well accepted by participants. Although no formal cut-off score is established for the CSQ-8, the mean score was near the upper end of the scale and notably higher than that reported in a large Norwegian validation study conducted in routine specialist outpatient care using the same digital format [41]. This comparison offers a relevant benchmark, as both studies were conducted in comparable clinical and methodological settings. The findings support the acceptability of the intervention and its potential suitability for implementation in routine mental health services.

There are several important limitations that should be noted. First, this was a pilot study, and the lack of a control group, along with the small sample size, limits how far the findings can be generalized. The naturalistic setting adds to the ecological validity, but it may also have introduced confounding factors that could have influenced the results. Since the study was a naturalistic study embedded in standard practice, we did not systematically record the number of patients who declined participation or were excluded prior to treatment. Because the study took place in a routine outpatient setting, assessments were not conducted independently, and we did not measure adherence to the treatment. We also did not control for additional treatments that participants may have received during or after the intervention. Although no new psychological treatment was introduced during the active treatment phase, some patients may have received follow-up care for comorbid conditions. This adds variability that future controlled studies should seek to address. Another limitation is the absence of a patient-reported outcome measure specifically for panic symptoms. This makes it difficult to compare clinician assessments with the patients’ own experiences. Future studies should include both clinician- and patient-rated outcomes to provide a more complete picture of treatment effects. It will be important to replicate these findings in larger and more controlled studies to better evaluate the effectiveness and potential of this treatment. Using independent assessors in future trials would also help reduce potential bias and improve methodological quality. In addition, the treatment should be tested in other clinical settings and cultural contexts. In conclusion, the present study indicates that the BrOST may be a promising approach for treating PD. This finding is in line with previous findings indicating that CBT can be delivered in a brief and cost-effective way without a significant reduction in treatment response.

## Data Availability

The data that support the findings of this study are not openly available due to reasons of sensitivity and are available from the corresponding author upon reasonable request. The data are stored in a controlled access database at Haukeland University Hospital.

## References

[1] Kessler RC, Chiu WT, Jin R, Ruscio AM, Shear K, Walters EE. The epidemiology of panic attacks, panic disorder, and agoraphobia in the National Comorbidity Survey Replication. Arch Gen Psychiatry. 2006;63(4):415–24. 10.1001/archpsyc.63.4.415 PMC1958997 16585471

[2] National Institute for Health and Care Excellence. Generalized anxiety disorder and panic disorder in adults. Clinical Guideline CG113. London; 2020.

[3] Öst LG, Ollendick TH. Brief, Intensive and Concentrated Cognitive Behavioral Treatments for Anxiety Disorders in Children: A Systematic Review and Meta-Analysis. Behav Res Ther. 2017;97:134–45. 10.1016/j.brat.2017.07.008 28772195

[4] Varma S. Ultra-short Term Cognitive Behaviour Psychotherapy for Panic Attacks. Malaysian Journal of Psychiatry. 1997;5(1):34–40.

[5] Westling BE, Öst L-G. Brief cognitive behaviour therapy of panic disorder. Scandinavian Journal of Behaviour Therapy. 1999;28(2):49–57.

[6] Craske MG, Maidenberg E, Bystritsky A. Brief cognitive-behavioral versus nondirective therapy for panic disorder. J Behav Ther Exp Psychiatry. 1995;26(2):113–20. 10.1016/0005-7916(95)00003-i 7593683

[7] Clark DM, Salkovskis PM, Hackmann A, Wells A, Ludgate J, Gelder M. Brief cognitive therapy for panic disorder: a randomized controlled trial. Journal of Consulting and Clinical Psychology. 1999;67(4):583. 10.1037//0022-006x.67.4.583 10450630

[8] Otto MW, Tolin DF, Simon NM, Pearlson GD, Basden S, Meunier SA, et al. Efficacy of d-cycloserine for enhancing response to cognitive-behavior therapy for panic disorder. Biol Psychiatry. 2010;67(4):365–70. 10.1016/j.biopsych.2009.07.036 19811776

[9] Otto MW, Pollack MH, Dowd SM, Hofmann SG, Pearlson G, Szuhany KL, et al. Randomized trial of d‐cycloserine enhancement of cognitive‐behavioral therapy for panic disorder. Depress Anxiety. 2016;33(8):737–45. 10.1002/da.22531 PMC5958622 27315514

[10] Davidsdottir SD, Sigurjonsdottir O, Ludvigsdottir SJ, Kvale G, Hansen B, Hagen K, et al. Preliminary effectiveness of the Bergen 4-day treatment for OCD in Iceland. Cognitive behaviour therapy. 2025;54(5):1–18. 10.1080/16506073.2025.2453722 39903681

[11] Skjold SH, Hagen K, Wheaton MG, Hjelle KM, Bjorgvinsson T, Hansen B. The effectiveness and acceptability of the Bergen 4-day treatment for adolescents with OCD: a replication and extension. BMC Psychiatry. 2024;24(1):148. 10.1186/s12888-024-05601-w PMC10882780 38383351

[12] Jelinek L, Serve A, Pampuch S, Scheunemann J, Schultz J, Miegel F, et al. Exploring the Peaks and Potholes: Understanding positive and negative effects of concentrated exposure treatment for obsessive-compulsive disorder. Journal of Obsessive-Compulsive and Related Disorders. 2024;43:100913.

[13] Launes G, Hagen K, Öst LG, Solem S, Hansen B, Kvale G. The Bergen 4-Day Treatment (B4DT) for Obsessive-Compulsive Disorder: Outcomes for Patients Treated After Initial Waiting List or Self-Help Intervention. Front Psychol. 2020;11:982. 10.3389/fpsyg.2020.00982 PMC7266968 32528372

[14] Kvale G, Hansen B, Hagen K, Abramowitz JS, Bortveit T, Craske MG, Franklin ME, Haseth S, Himle JA, Hystad S et al. Effect of D-Cycloserine on the Effect of Concentrated Exposure and Response Prevention in Difficult-to-Treat Obsessive-Compulsive Disorder: A Randomized Clinical Trial. JAMA Netw Open. 2020;3(8):e2013249. 10.1001/jamanetworkopen.2020.13249 PMC7426745 32789516

[15] Launes G, Laukvik IL, Sunde T, Klovning I, Hagen K, Solem S, et al. The Bergen 4-Day Treatment for Obsessive-Compulsive Disorder: Does It Work in a New Clinical Setting? Front Psychol. 2019;10:1069. 10.3389/fpsyg.2019.01069 PMC6534081 31164848

[16] Launes G, Hagen K, Sunde T, Ost LG, Klovning I, Laukvik IL, et al. A Randomized Controlled Trial of Concentrated ERP, Self-Help and Waiting List for Obsessive- Compulsive Disorder: The Bergen 4-Day Treatment. Front Psychol. 2019;10:2500. 10.3389/fpsyg.2019.02500 PMC6873786 31803089

[17] Hansen B, Kvale G, Hagen K, Havnen A, Öst LG. The Bergen 4-day treatment for OCD: four years follow-up of concentrated ERP in a clinical mental health setting. Cognitive behaviour therapy. 2019;48(2):89–105. 10.1080/16506073.2018.1478447 30088441

[18] Davíðsdóttir SD, Sigurjónsdóttir Ó, Ludvigsdóttir SJ, Hansen B, Laukvik IL, Hagen K, et al. Implementation of the Bergen 4-day treatment for obsessive compulsive disorder in Iceland. Clin Neuropsychiatry. 2019;16(1):33–8. PMC8650209 34908936

[19] Kvale G, Hansen B, Björgvinsson T, Bortveit T, Hagen K, Haseth S, et al. Successfully treating 90 patients with obsessive compulsive disorder in eight days: the Bergen 4-day treatment. BMC Psychiatry. 2018;18(1):323. 10.1186/s12888-018-1887-4 PMC6172736 30286745

[20] Hansen B, Hagen K, Ost LG, Solem S, Kvale G. The Bergen 4-Day OCD Treatment Delivered in a Group Setting: 12-Month Follow-Up. Front Psychol. 2018;9:639. 10.3389/fpsyg.2018.00639 PMC5943612 29774005

[21] Eide TO, Olsen T, Hansen H, Hansen B, Solem S, Hagen K. The Bergen 4-Day Treatment for panic disorder patients in a rural clinical setting: a long-term follow-up study. BMC Psychiatry. 2025;25(1):3. 10.1186/s12888-024-06445-0 PMC11697728 39748386

[22] Eide TO, Hansen B, Hjelle KM, Solem S, Wheaton MG, Björgvinsson T, et al. The bergen 4-day treatment for panic disorder: a longer-term follow-up. BMC Psychiatry. 2025;25(1):107. 10.1186/s12888-025-06527-7 PMC11806683 39920620

[23] Hjelle KM, Eide TO, Thorsen AL, Kvale G, Hagen K, Wheaton MG, et al. Long-term follow-up of the Bergen 4-day treatment for panic disorder during the COVID-19 pandemic. Cogent Psychology. 2024;11(1):2402603.

[24] Hjelle KM, Eide TO, Thorsen AL, Kvale G, Hagen K, Snorrason I, et al. The Bergen 4-day treatment for panic disorder: adapting to COVID-19 restrictions with a hybrid approach of face-to-face and videoconference modalities. BMC Psychiatry. 2023;23(1):570. 10.1186/s12888-023-05062-7 PMC10408203 37550696

[25] Eide TO, Hjelle KM, Saetre IU, Solem S, Olsen T, Skold RO, et al. The Bergen 4-day treatment for panic disorder: implementation in a rural clinical setting. BMC Psychiatry. 2023;23(1):305. 10.1186/s12888-023-04812-x PMC10152771 37127598

[26] Iversen HM, Eide TO, Harvold M, Solem S, Kvale G, Hansen B, Hagen K. The Bergen 4-day treatment for panic disorder: replication and implementation in a new clinic. BMC Psychiatry. 2022;22(1):728. 10.1186/s12888-022-04380-6 PMC9686090 36418989

[27] Hansen B, Kvale G, Hagen K, Hjelle KM, Solem S, Bø B, Öst LG. The Bergen 4-Day Treatment for Panic Disorder: A Pilot Study. Front Psychol. 2018;9:1044. 10.3389/fpsyg.2018.01044 PMC6029556 29997546

[28] Hansen B, Olsen Eide T, Reiråskag MA, Tjelle KA, Solem S, Hagen K. The Bergen 4-Day Treatment for Social Anxiety Disorder: A Pilot Study. BMC Psychiatry. 2024;24(145). 10.1186/s12888-024-05607-4 PMC10880199 38383324

[29] Clark DM, Ehlers A. An overview of the cognitive theory and treatment of panic disorder. Applied and preventive psychology. 1993;2(3):131–9.

[30] Sheehan DV, Lecrubier Y, Sheehan KH, Amorim P, Janavs J, Weiller E, et al. The Mini-International Neuropsychiatric Interview (M.I.N.I.): the development and validation of a structured diagnostic psychiatric interview for DSM-IV and ICD-10. J Clin Psychiatry. 1998;59(Suppl 20):22–33;quiz 34–57. 9881538

[31] Mordal J, Gundersen Ø, Bramness JG. Norwegian version of the Mini-International Neuropsychiatric Interview: feasibility, acceptability and test-retest reliability in an acute psychiatric ward. Eur Psychiatry. 2010;25(3):172–7. 10.1016/j.eurpsy.2009.02.004 19553089

[32] Shear MK, Brown TA, Barlow DH, Money R, Sholomskas DE, Woods SW, et al. Multicenter collaborative panic disorder severity scale. Am J Psychiatry. 1997;154(11):1571–5. 10.1176/ajp.154.11.1571 9356566

[33] Wuyek LA, Antony MM, McCabe RE. Psychometric properties of the Panic Disorder Severity Scale. Clinician‐administered and self‐report versions. Clin Psychol Psychother. 2011;18(3):234–43. 10.1002/cpp.703 21110405

[34] Furukawa TA, Katherine Shear M, Barlow DH, Gorman JM, Woods SW, Money R, Etschel E, Engel RR, Leucht S. Evidence‐based guidelines for interpretation of the Panic Disorder Severity Scale. Depress Anxiety. 2009;26(10):922–9. 10.1002/da.20532 PMC2760657 19006198

[35] Kroenke K, Spitzer RL, Williams JB, Lowe B. The Patient Health Questionnaire Somatic, Anxiety, and Depressive Symptom Scales: a systematic review. Gen Hosp Psychiatry. 2010;32(4):345–59. 10.1016/j.genhosppsych.2010.03.006 20633738

[36] Spitzer RL, Kroenke K, Williams JB, Lowe B. A brief measure for assessing generalized anxiety disorder: the GAD-7. Archives of internal medicine. 2006;166(10):1092–1097. 10.1001/archinte.166.10.1092 16717171

[37] Larsen DL, Attkisson CC, Hargreaves WA, Nguyen TD. Assessment of client/patient satisfaction: development of a general scale. Eval Program Plann. 1979;2(3):197–207. 10.1016/0149-7189(79)90094-6 10245370

[38] Lancaster GA, Dodd S, Williamson PR. Design and analysis of pilot studies: recommendations for good practice. Journal of evaluation in clinical practice. 2004;10(2):307–12. 10.1111/j..2002.384.doc.x 15189396

[39] Schafer JL. Analysis of incomplete multivariate data. Thousand Oaks, CA: Sage; 1997.

[40] Morris SB, DeShon RP. Combining effect size estimates in meta-analysis with repeated measures and independent-groups designs. Psychological methods. 2002;7(1):105–125. 10.1037/1082-989x.7.1.105 11928886

[41] Pedersen H, Havnen A, Brattmyr M, Attkisson CC, Lara-Cabrera ML. A digital Norwegian version of the client satisfaction questionnaire 8: factor validity and internal reliability in outpatient mental health care. BMC Psychiatry. 2022;22(1):671. 10.1186/s12888-022-04281-8 PMC9623922 36316661

[42] Shafran R, Clark DM, Fairburn CG, Arntz A, Barlow DH, Ehlers A, et al. Mind the gap: Improving the dissemination of CBT. Behav Res Ther. 2009;47(11):902–9. 10.1016/j.brat.2009.07.003 19664756

